# Full-length genome sequences of porcine epidemic diarrhoea virus strain CV777; Use of NGS to analyse genomic and sub-genomic RNAs

**DOI:** 10.1371/journal.pone.0193682

**Published:** 2018-03-01

**Authors:** Thomas Bruun Rasmussen, Maria Beatrice Boniotti, Alice Papetti, Béatrice Grasland, Jean-Pierre Frossard, Akbar Dastjerdi, Marcel Hulst, Dennis Hanke, Anne Pohlmann, Sandra Blome, Wim H. M. van der Poel, Falko Steinbach, Yannick Blanchard, Antonio Lavazza, Anette Bøtner, Graham J. Belsham

**Affiliations:** 1 DTU National Veterinary Institute, Technical University of Denmark, Lindholm, Kalvehave, Denmark; 2 IZSLER, Istituto Zooprofilattico Sperimentale della Lombardia e dell’Emilia Romagna “Bruno Ubertini”, Brescia, Italy; 3 ANSES–Laboratory of Ploufragan-Plouzané –BP 53, Ploufragan, France; 4 Université Bretagne Loire, Rennes, France; 5 Animal and Plant Health Agency, Department of Virology, Weybridge, Addlestone, Surrey, United Kingdom; 6 Wageningen BioVeterinary Research, Department of Virology, Lelystad, The Netherlands; 7 Friedrich-Loeffler-Institut, Federal Research Institute for Animal Health, Greifswald—Insel Riems, Germany; Oklahoma State University, UNITED STATES

## Abstract

Porcine epidemic diarrhoea virus, strain CV777, was initially characterized in 1978 as the causative agent of a disease first identified in the UK in 1971. This coronavirus has been widely distributed among laboratories and has been passaged both within pigs and in cell culture. To determine the variability between different stocks of the PEDV strain CV777, sequencing of the full-length genome (ca. 28kb) has been performed in 6 different laboratories, using different protocols. Not surprisingly, each of the different full genome sequences were distinct from each other and from the reference sequence (Accession number AF353511) but they are >99% identical. Unique and shared differences between sequences were identified. The coding region for the surface-exposed spike protein showed the highest proportion of variability including both point mutations and small deletions. The predicted expression of the ORF3 gene product was more dramatically affected in three different variants of this virus through either loss of the initiation codon or gain of a premature termination codon. The genome of one isolate had a substantially rearranged 5´-terminal sequence. This rearrangement was validated through the analysis of sub-genomic mRNAs from infected cells. It is clearly important to know the features of the specific sample of CV777 being used for experimental studies.

## Introduction

Porcine epidemic diarrhoea virus (PEDV) is the causative agent of an infectious disease, termed PED, which was initially recognized in the UK in 1971. The prototypic early European strain of PEDV was first characterized from infected pigs in 1978 and was named CV777 [1]. The virus is now classified as a member of the *Alphacoronavirus* genus within the family *Coronaviridae*. The CV777 strain has been propagated both in pigs and in Vero cell cultures. Like other coronaviruses, the virus particles have a characteristic “corona-like” morphology due to the presence of the surface exposed spike (S) protein. These viruses are enveloped and contain a positive sense RNA genome that is about 28,000 nucleotides (nt) in length; the complete genome sequence of the CV777 strain has been assembled [2] (Accession no. AF353511).

The disease is characterised by diarrhoea and vomiting leading to severe dehydration with high mortality among young piglets and hence severe economic losses. During the 1980’s and 1990’s, the disease spread within Europe and also in Asia where it has caused significant problems (reviewed in [3, 4, 5]). After this time, the disease declined within Europe and very few cases have been recorded there until recently. However, a new wave of PEDV infections occurred from 2010 onwards in China and adjacent countries [6] and the disease spread, for the first time, into the USA in 2013 [7]. Indeed, it appears that there have been two separate introductions of distinct PEDV strains into the USA [7, 8]. These viruses differ significantly within the sequence of the S gene (encoding the spike protein); these distinct strains have been classified as being within different genogroups (1b and 2b) based on the sequence of the S gene alone. These two strains of PEDV are also referred to as “INDEL” and “non-INDEL” respectively, reflecting the presence or absence of insertions and deletions within the S gene sequence (prototypic strains are: USA/OH851/2014, GenBank accession no. KJ399978, [9] and USA/Kansas29/2013, GenBank accession no. KJ645637.1, [10] respectively). However, neither form of nomenclature is entirely satisfactory. Indeed, both of these US variants of PEDV have been classified as being within genotype 2, based on complete genome sequences [5], while the early European strains, e.g. CV777, are in genotype 1.

Recently, new outbreaks of PED have occurred in various European countries including Germany, Italy, France, Portugal, Austria and Slovenia (see [11–16]). These new cases have nearly all been caused by viruses closely related to just one of the two US variants (closely related to OH851/2014). However, a case in Ukraine was caused by a strain most closely related to the Kansas29/2013 virus [17]. Full genome sequence analysis of the recent PED viruses circulating in the USA, China and Europe has been reported (see [7, 9, 11, 13, 17]).

It has been suggested that the OH851-like viruses cause less severe disease than the Kansas29/2013-like strains [9, 18]. However, it is apparent that the nature and severity of the disease can vary significantly even with apparently very similar viruses and thus other factors must also determine the outcome of the infection. These factors may include the age, immune status and general health status of the animals [19].

The CV777 virus has been widely distributed to different laboratories and has been passaged in both pigs and in cell culture. As a means of identifying the nature of the diversity within the CV777 strain (and its known close relatives, e.g. Br1/87, see [5, 20]), full genome sequence determinations were carried out in different laboratories on their “own” stock of this virus using Next Generation Sequencing (NGS) protocols. The resultant sequences have now been compared. Not surprisingly, each of the sequences obtained is different although closely related. The use of NGS on RNA isolated from infected cells also allowed evaluation of the sequences and relative levels of the various sub-genomic viral mRNAs. This analysis of these closely related viruses is important for interpreting the results from experimental studies performed with different samples of the “same” virus.

## Results

**Fig 1:**
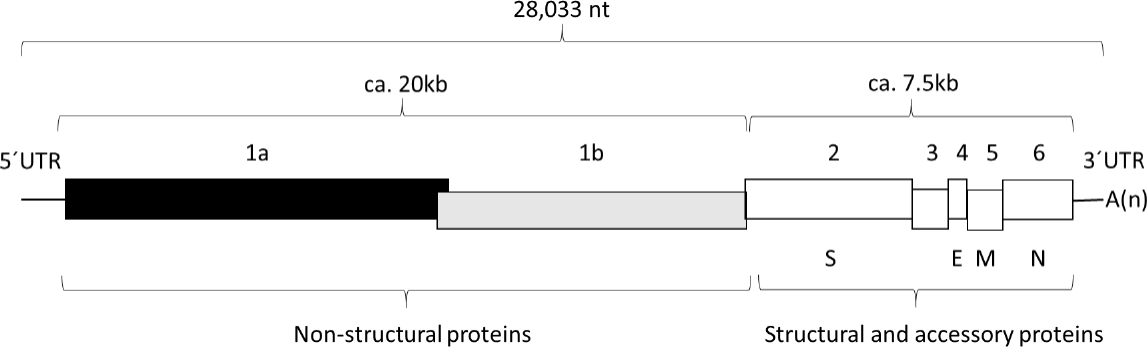
Representation of the genome organisation of PEDV. The open reading frames (ORFs) encoding the non-structural, structural and accessory proteins of PEDV are indicated. The genome contains untranslated regions (UTRs) at both the 5´- and 3´-termini. A poly(A) tail is present at the 3´-terminus.

All the sequences determined were very similar (>99% identical) to the reference CV777 sequence but each was unique (n.b., for the complete viral genome a 1% difference corresponds to 280 nt changes). Accession numbers for the sequences have been submitted to the ENA under the study accession no. PRJEB20818. Throughout the description of changes in the sequence, the numbering system used is that from the reference sequence of 28033 nt for CV777 [2] (Accession no. AF353511). The genome organization of PEDV is shown in Fig 1. The nt sequence changes for each of the different strains and the amino acid substitutions that result (when applicable) are listed in Table 1. Each of these six sequences lacked nt 72 and nts 82–85 compared to the reference sequence. The key features of the individual strains/sequences and for the separate regions of the genome (see Fig 1) are described separately below for each laboratory.

**Table 1:** Nucleotide and amino acid differences from the CV777 reference sequence AF353511.

Region	Nucleotide (nt)	AF353511 nt / aa	ANSES nt / aa	WBR nt / aa	DTU nt / aa		APHA nt / aa	IZSLER nt / aa	FLI nt / aa
5'-UTR	8	A	G		**	G			
	28	A			**	G			
	72	A	Del	Del	Del		Del	Del	Del
	82–85	TCCT	Del	Del	Del		Del	Del	Del
ORF1a/1b	3433	A / E							Del#
	3434	G / E							Del#
	3435	T / S							Del#
	3436	C / S							Del#
	3437	T / S							Del#
	3438	G / E		C / Q					Del#
	4102	T / V					Y / (A/V)		
	4284	G / G							A / G
	4767	G / A					T / S		
	5170	A / E		G / G					
	6607	T / L					C / Silent		
	7941	A / S					G / G		
	9179	C / Y					T / Silent		
	10715	T / A					Y / Silent		
	11103	G / A					Y / (A/S)		
	13594	T / V					G / Silent		
	13917	T / V	G / G		G / G		G / G		
	18113	A / M					G / V		
	20015	A / I					G / V		
Spike (S)	20887	G / G						A / S	A / S
	20984	T / I			C / T		C / T		
	21348	T / N					Y / Silent		
	21529	C / H					T / Y		
	21680	A / N					G / S		
	21805	C / P			T / S				
	22145	C / S						T / L	T / L
	22535	A / E			G / G				
	23771	C / A					T / V		
	23800	C / H					G / D		
	24416	C / T							A / N
	24757	T / Y					C / H		
Spike/ ORF3	24765–24816	*						Del	Del
ORF3	25201	A / I							Del
	25202	T / I					Del##		Del
	25203	T / I					Del##		Del
Env (E)	25655	G / S						K / (S/I)	
Mem (M)	25713	T / V		C / A					
	25992	G / R							A / H

* 52 nt deletion at Spike/ORF3 junction: TTTTGAAAAGGTCCACGTGCAGTG*A**TG*TTTCTTGGACTTTTTCAATACACGA. Stop codon of Spike and start codon of ORF3 indicated with underline and italics, respectively.

** the 5´-terminal region of the Br1/87 (DTU) sequence is rearranged upstream of nt 35.

#The 6 nt deletion modifies 3 codons and results in alteration of the encoded amino acid sequence from..NVESEV.. to..NVEV.., i.e. 2 amino acids are deleted.

## The loss of 2 nt results in the formation of a new termination codon that is predicted to result in the truncation of the ORF3 protein (see text for details).

Shared changes are highlighted in grey. Synonymous nt changes are indicated as “silent”. The nt identifier Y is for pyrimidines (C or T) while K denotes G or T.

### Sequence length and coverage of the different strains

#### a) DTU National Veterinary Institute (DTU-Vet)

A single contig of 28085 nt corresponding to the Br1/87 genome was assembled, *de novo*, from the sequence reads. Essentially, the same sequence was obtained from two independent RNA preparations (one sequence lacked the 5'-terminal 6 nt relative to the other). The average coverage across the genome was about 1250 reads per nt. There were just 4 nt changes (all non-synonymous) within the coding sequence compared to the reference sequence. Some more significant changes were detected near the terminus of the 5'-untranslated region (5'-UTR), these are described in more detail below.

#### b) Istituto Zooprofilattico Sperimentale della Lombardia e dell’Emilia Romagna (IZSLER)

A single contig of 27945 nt corresponding to the CV777 genome was assembled (note that compared to the reference sequence 24 nt are missing at the 5'-terminus and 7 nt at the 3'-terminus (mainly because of the PCR strategy used). The average coverage across the genome was about 953 reads per nt. In comparison to the reference sequence, in this sequence, there were 2 clear single nt changes and a mixture of bases at nt 25655 plus a single 52 nt deletion within the coding region.

#### c) ANSES–Laboratory of Ploufragan-Plouzané (ANSES)

A single contig of 28028 nt corresponding to the CV777 genome was assembled, *de novo*. The average coverage across the genome was 7103 reads per nt. There was just a single nt difference between this sequence and the reference sequence throughout the entire coding sequence. A single nt change (at nt 8) was observed in the 5'-UTR which was shared with the APHA sequence.

#### d) Friedrich-Loeffler-Institut (FLI)

A single contig of 27998 nt was assembled, *de novo*, for the V215-78 strain from 607383 reads, with a mean read length of 300 nt, giving an average coverage of 6508 reads per nt. Just 5 nt changes plus 3 separate deletions (3-52nt in length) were detected in the entire coding region compared to the reference sequence.

#### e) Animal and Plant Health Agency, Weybridge (APHA)

A single contig of 28026 nt corresponding to the genome of the APHA strain of CV777 was assembled, *de novo*, from the sequence reads. The average coverage across the genome was 122 reads per nt. About 20 nt changes from the reference sequence were present in the complete coding region and a single 2 nt deletion. Two single nt changes in sequence were also observed close to the 5'-terminus of the 5'-UTR (see below).

#### f) Wageningen BioVeterinary Research, Lelystad (WBR)

Sequence reads produced from the CV777 PEDV strain at WBR covered 99% of the PEDV genome with an average depth of 38 reads per nt. In total, some 282 nt of the reference sequence, divided over 11 stretches (≤ 59 nt), were not covered by the NGS reads (see S1 Table). Clearly, any changes in the sequence occurring at these positions are not described here. At 9 positions in the CV777 genome homogeneous nt differences (different in 100% of the reads)) were found between the assembled WBR CV777 consensus sequence and the CV777 reference sequence (see S2 Table). Three of these nt changes (at nt 3438, 5170 and 27713) were found in >25 reads and are included in Table 1, other changes are only supported by relatively few reads and were therefore excluded from Table 1. In addition, considerable sequence heterogeneity (but below the consensus level) was found at 10 positions in this CV777 genome (different in >33% but ≤50% of the NGS reads, with a depth of ≥4 reads; S3 Table).

### Sequence variations in the protein coding regions of the genome

#### i) Variation in the ORF1a/1b coding sequence

The IZSLER sequence is identical to the reference sequence throughout the entire length of ORF1a/1b (nt 297 to 20641, see Fig 1) while the ANSES and DTU sequence only differ from the reference sequence within this region at nt 13917 (see Table 1). This shared T to G change at nt 13917 results in a V to G amino acid substitution. The FLI sequence has a 6 nt deletion (nt 3433–3438) and a single silent mutation (at nt 4284), see Table 1. The 6 nt deletion within ORF1 affects 3 codons but just results in the loss of 2 amino acid residues and changes the predicted amino acid sequence from …NVESEV… to …NVEV…. The APHA sequence has up to 11 nt changes but at 3 of these positions, the sequencing indicated a mixed population (C or T, one of which is present in the reference sequence at 2 of these positions). Several of the mutations were silent while the non-synonymous nt change at nt 13917 was shared with the ANSES and DTU sequences as indicated above. The WBR sequence contained 2 non-synonymous nt changes within ORF1 compared to the reference sequence.

#### ii) Variation in the S protein coding sequence

**Fig 2:**
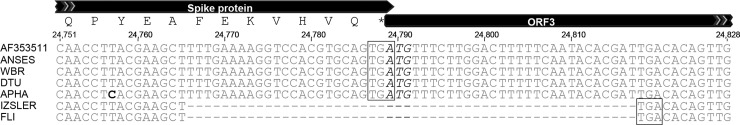
Identical deletions at the junction of the S protein and ORF3 protein coding regions in two PEDV strains. The PEDVs sequenced by IZSLER and FLI have identical 52 nt deletions which removes the usual termination codon (marked in a box). This can be predicted to result in the removal of 7 amino acids from the C-terminus of the spike protein and the use of a different termination codon (marked in another box) but no extraneous residues will be added to the S protein. In addition, this deletion removes the initiation codon for ORF3 (indicated in italics), which can be expected to result in complete loss of ORF3 protein expression. A single, non-synonymous, nt change (T24757C, resulting in a Y to H substitution) is present in the APHA sequence and is indicated in bold type.

The spike protein coding sequence of the ANSES strain is identical to the reference CV777 sequence. Within this region, the IZSLER sequence varies at 2 nt positions from the reference sequence and these changes are both shared with the FLI sequence (see Table 1) but the FLI sequence also has a third, non-synonymous change (at nt 24416). In addition, just the IZSLER and FLI sequences share a 52nt deletion (nt 24765–24816) at the extreme 3'-terminus of the spike protein coding sequence which removes the usual termination codon and also the initiation codon for ORF3 (see below). It is, therefore, predicted that the spike protein that is produced by these strains lacks the usual seven C-terminal amino acid residues (FEKVHVQ) (see Fig 2). A new in-frame termination codon is produced at the new junction of the sequences created by the deletion and hence no extraneous amino acids will be present in the protein.

The Br1/87 spike protein coding sequence (from DTU) varies at 3 positions from the reference sequence (see Table 1), these non-synonymous changes are each distinct from the IZSLER/FLI changes in CV777 but one of them is shared with one of the 7 nt changes (only 1 of which is synonymous) in the APHA sequence.

#### iii) Variations in ORF3

**Fig 3:**
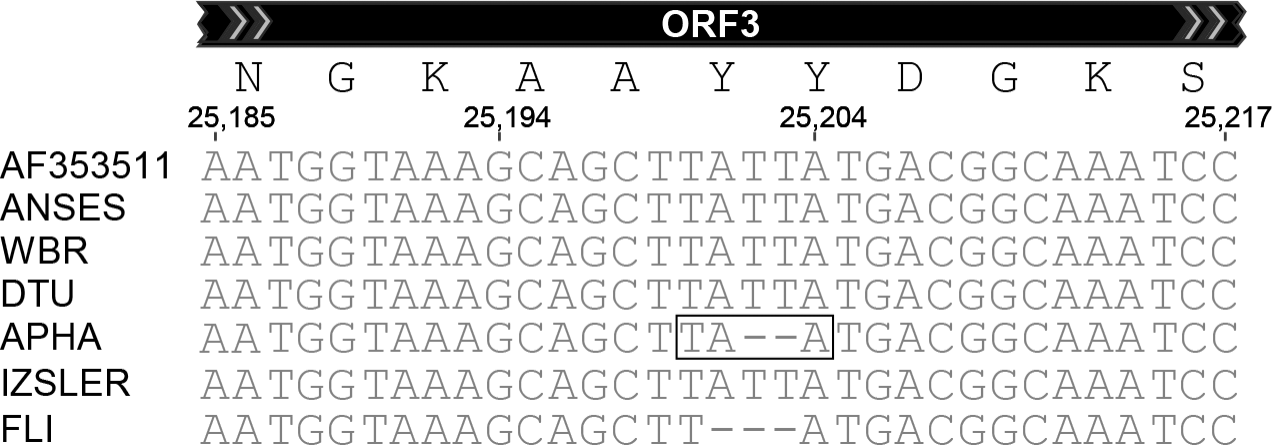
Short, internal, deletions within the ORF3 coding region. A 2 nt deletion within the ORF3 coding region of the APHA strain produces a new termination codon (TAA, boxed) and hence the ORF3 protein is predicted to be severely truncated (see text). A 3 nt deletion in the FLI sequence should result in the loss of a single amino acid but since the initiation codon of ORF3 is lost in this virus (see Fig 2) then this change is unlikely to have any consequence.

The 52 nt deletion in the IZSLER and FLI sequences described above, also removes the initiation codon for the ORF3 from these viruses and thus no expression of a functional ORF3 protein can be expected (Fig 2). There is also a 3 nt deletion (nt 25201–25203) in the residual ORF3 coding sequence within the FLI strain which would make for an in-frame deletion but since the initiation codon is lost then this is probably not significant. Interestingly the APHA sequence lacks just 2 nt (nt 25202–25203) at this point (Fig 3). The presence of this 2 nt deletion has been confirmed using Sanger sequencing of a conventional RT-PCR amplicon including this region. The deletion results in the production of a new, in-frame, stop codon and hence premature termination of the ORF3 protein (Fig 3). It is predicted that the truncated ORF3 product is 137 aa in length, compared to 224 aa for the reference sequence. No differences from the reference sequence were detected in the ORF3 coding sequence in the strains used at ANSES, WBR and DTU.

#### iv) Variation in the E and M protein coding sequences

The ANSES, DTU and APHA sequences were identical to the reference sequence throughout this region. The IZSLER sequence was heterogeneous at nt 25655 (within the E coding region) while the FLI sequence had a single coding change at nt 25992 (within the M coding region). The WBR sequence had a separate, single nt change within the M protein coding region (Table 1).

### Sequence variation in the untranslated regions of the PEDV genome

#### i) 3´-untranslated region (3´-UTR)

No differences were detected in the 3'-UTRs in any of the sequences but the extent of sequence generated varied to some degree (as indicated above).

#### ii) 5'-untranslated region (5'-UTR)

**Fig 4:**
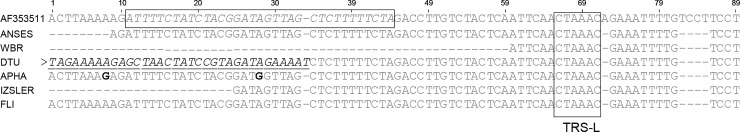
Alignment of the 5´-terminal sequences of the PEDV genomes. From nt 34 of the AF353511 reference sequence the different sequences align well but the reference sequence contains an insertion of one nt (nt 72) and a duplication of 4nt (see nt 82–89) that are absent in each of the other sequences. Due to the sequencing strategies used not all the sequences are complete. The Br1/87 (DTU) terminal sequence is extensively rearranged (see text for details) and contains a region (in black italics) that is the “reverse complement” of part of the reference sequence (boxed italics). Two single nt changes were found in the APHA sequence, marked in bold type, one change was shared with the ANSES sequence. The TRS-L is also indicated. The > symbol indicates that additional sequence is present (but not shown here) at the 5´ terminus in the DTU sequence.

There is some heterogeneity in the extreme 5'-termini of the different sequences. In part, this reflects differences in the NGS strategies and maybe also the smaller number of reads at the termini of the sequences. From nt 34 onwards, there is general agreement between the sequences and the core of the transcription regulatory sequence (TRS-L) motif (CUAAAC, nt 66–71) is conserved in all the sequences (see Fig 4). However, nt 72 (an A) in the reference CV777 sequence (AF353511) is missing in all the other sequences, this nucleotide is immediately adjacent to the core sequence of the TRS-L. In addition, there is a TCCT motif (nt 82–85) duplicated in the reference CV777 sequence (AF353511) whereas there is clearly just one copy of this motif in the 6 distinct sequences generated in this study (Table 1, see Fig 4). The APHA sequence also includes 2 single nt changes in the 5'-UTR, at nt positions 8 (A to G) and 28 (A to G), the same change at nt 8 was also found in the ANSES sequence (Table 1). Evidence for a more extensive rearrangement of the 5'-terminal sequence was found at the extreme end of the Br1/87 sequence (from DTU-Vet), this is discussed separately below.

### Evidence for genome rearrangement in the 5' UTR of the Br1/87 strain of PEDV

**Fig 5:**
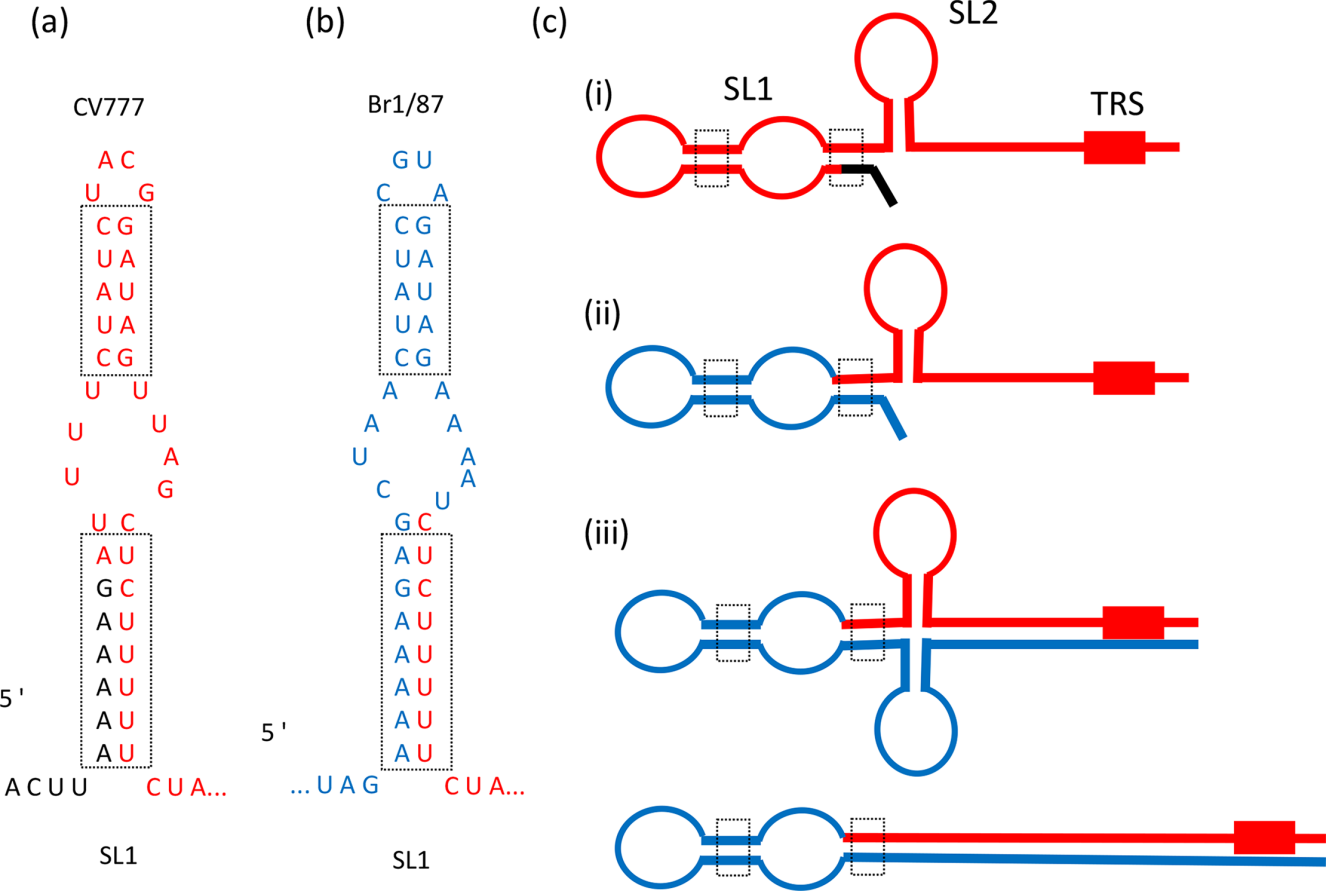
Secondary structure at the 5´-termini of the PEDV RNA genome. Multiple stem-loop (SL) structures have been predicted to be present in the 5´-terminal region of the PEDV genome [21]. The structure, termed SL1, closest to the terminus, in the CV777 reference strain is indicated in panel (a). A similar structure, predicted for the consensus Br1/87 sequence, is shown in panel (b). Note, the sequence marked in blue is the “reverse complement” of the sequence marked in red from panel (a). The conserved stems within SL1 are boxed but the apical loop region is distinct. In panel (c), a comparison of secondary structure predictions derived by M-fold for the CV777 5´-terminal sequence (i) and the consensus Br1/87 sequence (ii), are shown. The structures have the same apical loops as the SL1 structure shown in panels (a) and (b). The extended sequences (in blue), that include self-complementary regions extending beyond the TRS are indicated in panel (c) (iii). The complementary sequences (e.g. the sense and anti-sense SL2 regions) can simply base pair to each other to form an extended stem-loop structure that can be present at the 5´-terminus of the genomic and sub-genomic mRNAs (see also Fig 6).

From the *de novo* genome assembly of the Br1/87 strain of PEDV, it was noted that the 5' terminal sequence of the Br1/87 sequence was elongated at its 5' terminus and poorly matched to the 5´-terminal sequence of the reference strain (see Fig 4). However, by nt 34 of the reference sequence, close similarity with the Br1/87 sequence is established. The nt 1–33 of the reference sequence are absent in the Br1/87 sequence but the “reverse complement” of nt 11–44 are present within the *de novo* assembled Br1/87 sequence (see Figs 4 and 5A and 5B). By mapping the 5´ terminal sequence of Br1/87 from two independent libraries of sequence reads, prepared from RNA harvested at 24h or 48 h post infection (hpi) from within infected cells, it was found that 74 and 119 different reads corresponded to this modified structure in the genomic RNA (S4 Table). This “reverse complement” sequence was also identified in the leader sequences of the sub-genomic RNAs that allow the expression of the 6 different downstream ORFs. However, when the sequence reads were mapped onto the reference CV777 consensus 5'-terminal sequence then just 8 and 28 reads in the two different libraries matched the reference CV777 sequence.

**Fig 6:**
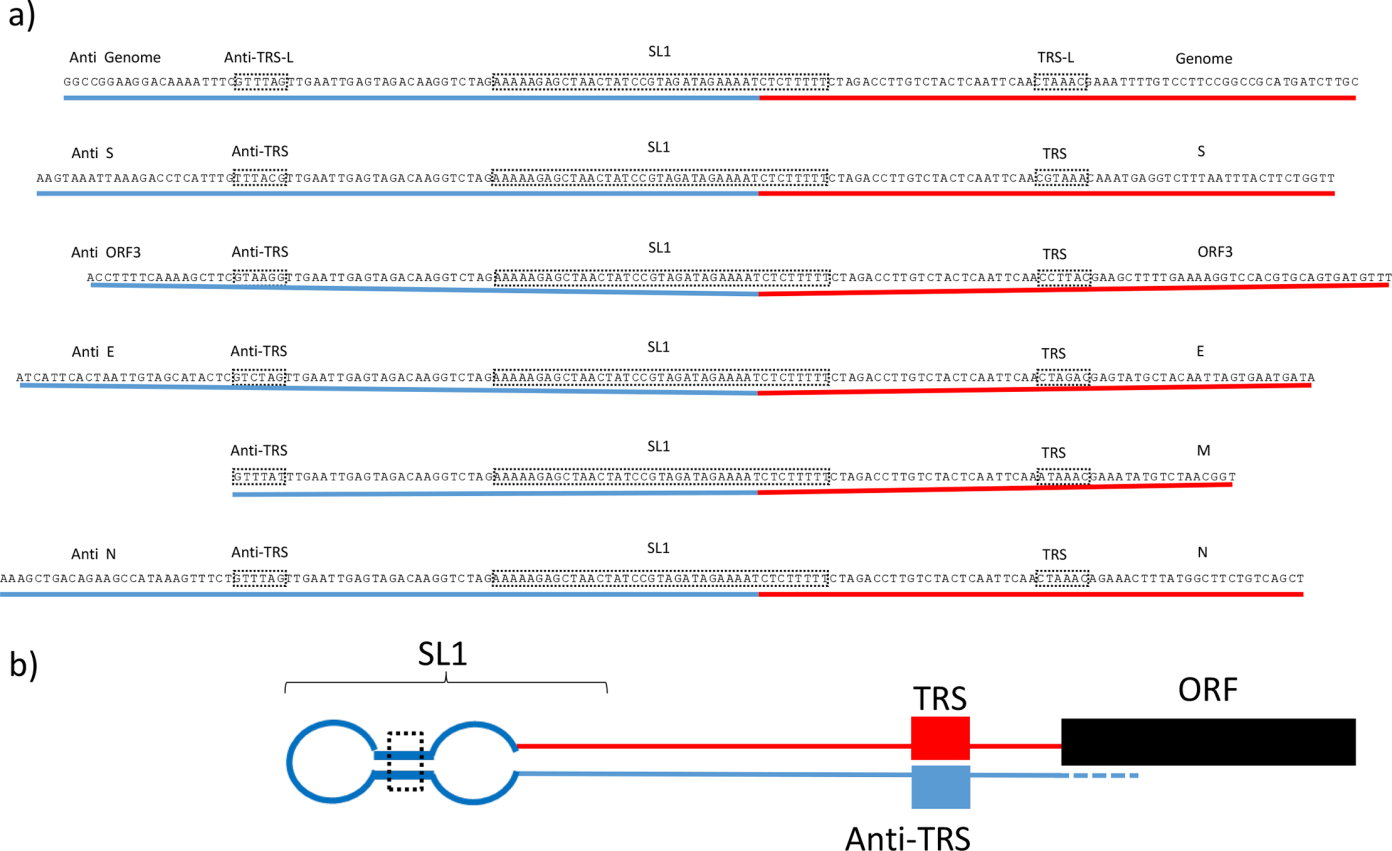
Self-complementary sequences present at the 5´-terminus of genomic and sub-genomic RNAs isolated from Br1/87 infected cells. Panel (a) The sequences of selected reads corresponding to the 5´-terminus of the genomic RNA and the 5 different sub-genomic mRNAs are shown. Each sequence shown is representative of multiple reads identified within the total collection of reads. The colour coding of different regions of the RNA is the same as that used in Fig 5. Panel (b). Representation of the shared structure at the 5´-termini of the genomic and sub-genomic RNAs. The self-complementary regions within each RNA are indicated by red and blue lines. The locations of the SL1, the TRS and its complement (Anti-TRS) are indicated by boxes.

Additional mapping of the Br1/87 sequences also identified reads that contained the “reverse complement” of nt 11 up to approx. nt 88 of the reference sequence (i.e. beyond the TRS). This indicates that the 5´-terminal sequence of the Br1/87 RNA contains a modified leader sequence that spans from nt 11 and into stem loop 4 (SL4, see [21]), of the reference sequence (Fig 6). The presence of this extensive self-complementary sequences means than a large stem-loop structure can be formed at the 5´-terminus of the genomic RNA (see Fig 5(C)). Interestingly, within the minority of reads that correspond to the reference CV777 sequence there is a single nt change (A to G) corresponding to nt position 8 in the reference sequence that matches one of the two changes observed in the APHA and ANSES CV777 sequences (see above). Thus, it seems that there is heterogeneity within the Br1/87 virus population and that the majority of genomes and the sub-genomic mRNAs produced within infected cells have a rearranged 5'-UTR compared to the consensus CV777 genome. Clearly, the presence of the “reverse complement” sequence indicates that the modified sequence is not just random rearrangement. Furthermore, interestingly, it is possible to derive a secondary structure model for the 5'-terminal stem loop structure of the Br1/87 sequence (SL1, see [21]) that appears similar to that predicted for the CV777 sequence and could be an alternative for the usual SL1 structure (see Fig 5A and 5B). In particular, the stem regions are completely conserved even though the upper stem in the Br1/87 sequence is the “reverse complement” of the CV777 SL1 sequence. However, the terminal loop sequence of the predicted SL1 is completely different, (CUACGG in CV777 and CCGUAG in Br1/87).

Mapping of other sequence reads derived from the Br1/87 virus infected cells which correspond to sub-genomic mRNAs indicated that extensive 5´-terminal stem-loop structures will also be present on the sub-genomic mRNAs as well (Fig 6).

## Discussion

Each of the PEDV sequences generated in this study are closely related (≥ 99% identity, excluding the sequence gaps in the WBR sequence) to each other and to the reference CV777 sequence. The ANSES sequence is the most closely related to the reference sequence, there is only a single nt difference in the whole coding region (ca. 27.5 kb, see Fig 1) and this results in a single amino acid substitution within the large ORF1 (ca. 20kb), and this change is shared with the DTU and APHA sequences (Table 1). It is noteworthy that the ANSES sequence was derived from a virus sample that had not been grown in cell culture but was derived directly from intestinal contents of an infected piglet that were collected in 1982.

Overall, the spike protein coding region (ca. 4.2kb) appears to be the most variable region between the various strains, there are more changes in this portion of the genome than in the whole of ORF1 (which is about 5 times larger) and many of these changes are unique to individual strains (see Table 1). In two of the strains (from FLI and IZSLER, which appear very closely related to each other), the coding region for the C-terminal 7 residues of the spike protein is lost (due to an identical 52nt deletion in each sequence) but the consequences of this change for the properties of the protein are not known. Other variants of PEDV containing large deletions within the spike protein coding sequence have been described [22].

The greatest diversity in protein expression is predicted for the ORF3 product. For the FLI and IZSLER strains, the initiation codon for ORF3 has been deleted (as part of the 52nt deletion that removed the usual termination codon of the S gene, see Fig 2) and thus the correct protein product cannot be expressed. In the APHA sequence, a 2 nt deletion within the ORF3 coding region is predicted to cause premature termination of the protein (Table 1 and Fig 3). Thus, 3 of the strains studied here do not seem to be able to produce an intact and functional ORF3 product. It has been noted previously that the ORF3 coding region of the CV777 strain is highly variable (see [2]). The function of this ORF in PEDV is not yet clear.

The APHA sequence has several silent mutations (mainly in ORF1), however, it is apparent that, in general, there are rather few silent mutations between the strains. Some of these were identified as sequence ambiguities, i.e. Y, (either C or T), and thus are not changed in all the reads (Table 1).

Within the 5'-UTR, the reference sequence is unique in having an extra nt (nt 72), this is located adjacent to the core sequence of the TRS-L motif. There is also an apparent duplication of nt 82–85 in the reference sequence compared to all the other strains studied here. It may be that these differences correspond to old sequencing errors or may be due to sequencing of cDNAs corresponding to sub-genomic mRNAs but they could be true changes of unknown significance. It is noteworthy that the presence of nt 72 and the duplication of nt 82–85 observed in the reference strain sequence (see Fig 4) are also lacking in other closely related PEDV sequences (e.g. the CV777 vaccine strain (Acc. No. KC189944)) but these features are present in the LZC sequence (Acc. No. EF185992, see [23]).

The Br1/87 virus was found to contain a significantly different sequence at the 5'- terminus of the genome compared to the others. From the consensus sequence derived from the NGS data, it appears that some rearrangement of these terminal sequences has occurred in most of the genomes. This does not appear to be a simple artefact since the derived sequence includes the “reverse complement” of a part of the reference virus sequence. It is possible to predict an alternative, but closely related SL1 structure for the Br1/87 sequence that maintains the sequence of the two stems of the CV777 SL1 (c.f. Fig 5(A) and 5(B)) but has a distinct apical loop. Recent studies have revealed conservation of this SL1 loop structure and an essential role in virus replication [21, 24]. Additional support for this rearranged sequence was derived from the sequence of reads corresponding to sub-genomic mRNAs (see Fig 6). It is currently unclear what effect the presence of the rearranged 5´-terminal sequence in the Br1/87 genomic RNA and sub-genomic mRNAs will have on the function of these transcripts. It can be envisaged that the presence of a stable hairpin structure at the 5'-termini of these RNAs would be strongly inhibitory to cap-dependent translation initiation (see [25, 26]) and thus the production of viral proteins can be expected to be low. Furthermore, it is interesting to note that the proportion of sub-genomic mRNA reads containing the rearranged leader fused to the S protein coding region is unexpectedly high (see S4 Table). This is not the case for the reads corresponding to the sub-genomic mRNAs including the CV777 5´-terminal sequence, which is consistent with previous data (e.g. see [27]). This requires further study but it may suggest that the modified leader becomes part of the transcript that encodes the S protein with a higher efficiency than to the other sub-genomic mRNA sequences. Thus, the ratio of viral proteins produced could also be affected.

It may be that the viral RNAs with the rearranged leader sequence act as defective interfering (DI) genomes and that only the minor population (similar to the CV777 sequence) of the cell culture grown Br1/87 viral RNA is able to function independently. The production of coronavirus DI genomes within infected cells is well documented (see [28]).

## Material and methods

### Viruses

The sources of the CV777 strain (or closely related strain) differed between the various laboratories, as did the protocols for generating the complete virus genome sequences. These details are described, in brief, from each contributing laboratory.

#### a) DTU-Vet

The virus strain used at DTU-Vet is termed Br1/87. Intestinal material from an infected pig (designated PED 1/87) was initially obtained from the Central Veterinary Laboratory, Weybridge, U.K, (now APHA), in 1987, and then passaged in pigs (twice) before being isolated in Vero cell culture [29] (note, Vero cells (from ATCC) are an established line of African green monkey kidney cells). This virus isolate has been used previously for sequencing studies [20] and referred to as the “British PEDV isolate” (Br1/87). This Vero cell culture grown Br1/87 virus causes typical clinical signs of PED in inoculated pigs [30].

For the NGS, preparations of RNA were made from Br1/87-infected Vero cells, at both 24 and 48 hpi, when CPE was apparent, using a MagnaPure robot and the total nucleic acid isolation kit (Roche). It should be noted that since the RNA was isolated from the infected cells then these samples contain both the full-length genomic RNA and also the sub-genomic PEDV mRNAs. In brief, DNA depletion was performed on the extracted nucleic acids using DNAse I and then 1^st^ strand cDNA was synthesized using SuperScript III (Invitrogen) using random hexamer primers (pN_6_). The RNA was removed from the 1^st^ strand cDNA using RNAse H and 2^nd^ strand synthesis was achieved using the NEBNext mRNA second strand synthesis kit (New England Biolabs). The ds cDNA was purified (GeneJET PCR purification kit) and quantified. Samples were sequenced at the DTU Multi-Assay Core (DMAC, Kgs. Lyngby, Denmark) using the Nextera-XT DNA library Preparation kit and a MiSeq with Reagent Kit v3 600 bp (Illumina Inc, San Diego, CA, USA). The sequence reads were analysed using CLC Genomics (Qiagen) and Geneious software (v. 6.1.8; Biomatters).

#### b) IZSLER

The virus isolate used at IZSLER is designated CV777. A lyophilized virus sample, 5^th^ passage in Vero cells, was provided, in 2003, from the University of Gent that had received it from Switzerland. The CV777 was then grown in Vero cells until the 9^th^ passage that was used for the NGS. There is no information available about the virulence of this strain in pigs. RNA was extracted from 200 μl of virus culture supernatant using the TRIzol method (QIAzol Lysis Reagent, QIAGEN, Hilden, Germany) according to the manufacturer’s instructions and was resuspended in 10 mM Tris-HCl, 1 mM EDTA, pH 8. Reverse transcription was performed with this total RNA using the SuperScript® III First-Strand Synthesis SuperMix (Invitrogen, CA, USA) using random hexamer primers, according to the manufacturer’s protocol. The complete genome sequence was assembled following amplification of 7 overlapping long PCR products covering the whole genome from the cDNA using the AccuPrime™ Taq DNA Polymerase High Fidelity kit (Invitrogen) according to the manufacturer’s protocol. The primers used are described in S5 Table. The PCR products were purified by Nucleospin® gel and PCR clean-up (Macherey-Nagel, Düren, Germany) and quantified. The sequencing libraries were prepared using the Nextera-XT DNA library Preparation kit (Illumina Inc.), purified according to the manufacturer’s protocol and sequenced on an Illumina MiSeq using the Miseq Reagent Kit v2, 250-cycle paired-end run (Illumina Inc.). Reads were analyzed using the SeqMan module of DNASTAR software package (Lasergene, Madison, USA).

#### c) ANSES

The CV777 strain was obtained from Dr. M. B. Pensaert in the early 1980’s. The virus was amplified by oral infection of specific pathogen free (SPF) piglets in 1982. An intestinal loop was inserted into the piglets and the intestinal contents were collected and then stored at -80°C. This strain was not amplified in cultured cells. The viral stock stored at -80°C since 1982 has been inoculated recently by the oral route into SPF piglets at 3 weeks of age (10^8^ genome copies/pig). The experiment was carried out in the air-filtered level 3 biosecurity facilities of ANSES in accordance with the European and French regulations on animal welfare. The protocol for this experiment was approved by the Ethics Committee registered under number #16 by the French Ministry of Research (No.03596.03). Two piglets were euthanized at 4 days post-inoculation and showed macroscopic lesions typical of PED and PEDV genome was detected in affected tissues.

For NGS sequencing, 1 mL of the intestinal contents from 1982 was homogenized in 9 mL of phosphate saline buffer (PBS). One mL of this suspension was DNase/RNase treated and then the total RNA was extracted from the treated material using Trizol reagent (Trizol LS, Life Technologies). The total RNA extract (20–70 ng) was treated with DNase using the TURBO DNA-free kit (Ambion) then depleted of ribosomal RNA using the Low Input RiboMinus Eukaryote System Kit (Ambion). This DNA/rRNA depleted RNA sample was then converted into representative cDNA libraries, using the Ion Total RNA-Seq Kit (Life Technologies) according to the supplier’s instructions with two modifications: RNA was fragmented with RNase III for 1 min at 37°C and the fragmented RNA was hybridized and ligated to adaptors overnight at 16°C. The resulting cDNAs were amplified by 18 cycles of PCR with IonXpress RNA-Seq Barcode Primers (Life Technologies). The size distribution of the library was assessed for quality with the High Sensitivity DNA kit (Agilent). 30–60% of fragments were >160 bp, thus validating the quality of the library. Sequencing of the cDNA library started with 10pmol of the amplified library using the Ion One Touch 2 system (Life Technologies). Sequencing was performed using the Ion Proton Sequencer and an Ion PI Chip v2 (Life Technologies).

Sample reads were cleaned with Trimmomatic software [31] and aligned to the PEDV CV777 (Accession no. AF353511) complete genome using tmap (Torrent Suit 4.0.2, https://github.com/iontorrent/TMAP) to evaluate coverage depth for each sample, Mira assembly [32] was ran on uncleaned, downsampled reads to a mean coverage depth of 80 after best kmer size estimation by kmergenie [33]. Spades assemblies [34] were performed after Trimmomatic cleaning.

Contigs were aligned and reordered to the reference sequence with Mauve [35]. Unrelated contigs were removed. Viral contigs not matching the reference were kept. Ambiguous nucleotides in the *de novo* assembly contigs were manually curated by visualizing read alignments in Tablet [36].

#### d) FLI

The PEDV strain V215-78 was from the FLI virus collection, it was obtained from within Europe in 1978 and was grown in Vero cells. There is no information available about the virulence of this strain in pigs.

RNA was extracted from infected Vero cell culture supernatants using Trizol Reagent (LifeTechnologies, Darmstadt, Germany) in combination with the RNeasy Mini Kit (Qiagen, Hilden, Germany) and DNase digestion was performed on the spin column. Further concentration and cleaning was achieved using Agencourt® RNAClean® XP magnetic beads (Beckman Coulter, Fullerton, USA). The RNA yield was determined using the Nanodrop ND1000 UV spectrophotometer (Peqlab, Erlangen, Germany). This RNA was used as the template for cDNA synthesis using a cDNA Synthesis System (Roche, Mannheim, Germany) and fragmented using a Covaris M220 Focused-ultrasonicator (Covaris, Brighton, United Kingdom) aiming at a target size of 500 bp. The fragmented cDNA was then converted into barcoded libraries using Illumina compatible adapters (Bioo Scientific Corp., Austin, USA) using a SPRI-TE library system (Beckman Coulter) with SPRIworks Fragment Library Cartridge II (for Roche FLX DNA sequencer; Beckman Coulter) without size selection. Upper and lower size selection was performed manually using Agencourt® AMPure® XP magnetic beads (Beckman Coulter) with a target peak size of 670 bp. These libraries were then quality and quantity checked using a Bioanalyzer 2100 (Agilent Technologies, Böblingen, Germany) and Kapa Library Quantification Kit for Illumina (Kapa Biosystems, Cape Town, South Africa) on a Bio-Rad CFX96 Real-Time System (Bio-Rad Laboratories, Hercules, USA). Sequencing was performed with an Illumina MiSeq instrument with MiSeq reagent Kit v3 for 600 cycles (Illumina, San Diego, USA).

Sequence assembly, the subsequent mapping of the raw sequence data, and the analysis of the resulting sequences were achieved with the Genome Sequencer software suite (v. 3.0; Roche) and the Geneious software suite (v. 8.1.3; Biomatters).

#### e) APHA

The virus strain used at APHA is also designated CV777. This strain was initially obtained from Dr M. B. Pensaert [1] and was passaged in pigs and later in Vero cells. There is no information available about the virulence of this strain in pigs.

For the NGS, RNA was isolated from CV777-infected Vero cells when CPE was apparent using TRIzol™ (Invitrogen). The extracted PEDV RNA was subjected to DNase digestion and used as the template for cDNA generation using the cDNA Synthesis System (Roche) which was subsequently used for the preparation of sequencing libraries using the Nextera XT kit (Illumina Inc.). Paired end sequencing was performed on an Illumina MiSeq. The consensus sequence was obtained by *de novo* assembly using Velvet 1.2.10 and re-evaluated using the templated genome assembly function of the SeqMan NGen v13 software (DNASTAR Inc. Madison, USA) and the consensus sequence was generated using the SeqMan Pro v13 software.

#### f) WBR

The virus strain CV777 was obtained from Pensaert & de Bouck in the late 1970’s and induced diarrhea in experimental pigs [1] but there is no information about the virulence in pigs of the cell-grown material. The virus was grown in Vero cells (see [5]) using serum-free medium supplemented with 10 μg/ml of trypsin [37]. The medium was harvested when 50% of the cells displayed CPE (4 days post infection), centrifuged at 4000 x g for 10 min to remove cell debris and concentrated 30-fold using a 30K Amicon Ultracel centrifugal filter (Amicon, Fisher Scientific, Landsmeer, The Netherlands). From this concentrated fraction, RNA was isolated using Trizol LS (Invitrogen). After DNase I digestion, a cDNA library for MiSeq sequencing was prepared as described [38]. Briefly, 1^st^ strand and 2^nd^ strand cDNA was synthesized using a degenerate primer with a sequence tag (GTTTCCCAGTCACGATA-N9) and linearly amplified by PCR using the tag primer GTTTCCCAGTCACGATA (annealing to 5' terminal sequence of the 2^nd^ strand cDNA). Fragments under 200 bp in length were removed and the integrity and size distribution of the cDNA library was determined with an Agilent 2100 Bioanalyzer using the High Sensitivity DNA Kit (Agilent) and shown to meet the quality criteria for MiSeq sequencing recommended by Illumina. PEDV-specific reads were extracted from MiSeq sequence data files by alignment to the reference CV777 sequence (AF353511.1) using blastn analysis. PEDV-reads were assembled to contigs and sequence differences between the *de novo* PEDV-sequence and the reference CV777 sequence (AF353511.1) were visualized using the ‘‘Tablet” program [39].

### RNA secondary structure prediction

The secondary structure of the 5´-terminal region of the viral RNA was predicted using M-fold [40].

## Supporting information

S1 Table Missing sequences (i.e. zero coverage) in WBR sequence compared to the reference CV777 sequence (AF353511.1). (DOCX)

S2 Table Homogenous nt differences in WBR CV777 sequence compared to reference CV777 sequence (in 100% of the NGS reads). (DOCX)

S3 Table Heterogeneous sequence differences in WBR CV777 sequence compared to reference CV777 sequence (heterogeneity present in >33% and ≤50% of the NGS reads). (DOCX)

S4 Table Number of read variants (with 100% identity) present in two independent Br1/87 RNA preparations from virus-infected cells made at 24h and 48h post infection (hpi). For each preparation the total collection of reads were mapped separately to the Br1/87 leader/TRS-L and the CV777 leader/TRS-L.
(DOCX)

S5 Table Oligonucleotide primers used for the production of overlapping amplicons at IZSLER. (DOCX)
