# Supplementary material for: Full-length genome sequences of porcine epidemic diarrhoea virus strain CV777; Use of NGS to analyse genomic and sub-genomic RNAs
Source: PLoS One. 2018 Mar 1;13(3):e0193682. doi: 10.1371/journal.pone.0193682 (PMC5832266; doi:10.1371/journal.pone.0193682)
Supplement: S1 Table — (DOCX) [file pone.0193682.s001.docx]

**Supplementary Information for Rasmussen et al., Full-length genome sequences of porcine epidemic diarrhoea virus strain CV777; use of NGS to analyse genomic and sub-genomic RNAs.**

| **S1 Table.**  Missing sequences (i.e. zero coverage) in WBR sequence compared to the reference CV777 sequence (AF353511.1) | | | | | | | | | |
| --- | --- | --- | --- | --- | --- | --- | --- | --- | --- |
|  |  | | |  | |  | |  | |
| nt start | | nt end | | | Missing sequence length | |  | |  |
| 1 | | 59 | | | 59 | |  | |  |
| 72 | | 72 | | | 1* | |  | |  |
| 82 | | 85 | | | 4* | |  | |  |
| 286 | | 382 | | | 97 | |  | |  |
| 541 | | 553 | | | 13 | |  | |  |
| 997 | | 1012 | | | 16 | |  | |  |
| 4682 | | 4714 | | | 33 | |  | |  |
| 5053 | | 5060 | | | 8 | |  | |  |
| 7325 | | 7357 | | | 33 | |  | |  |
| 15859 | | 15866 | | | 8 | |  | |  |
| 19124 | | 19133 | | | 10 | |  | |  |
|  | | Total (nt) | | | 282 | |  | |  |
|  | | |  |  | |  | |  | |

*These nt were also absent in other sequences obtained in this study (see Table 1).
