# Supplementary material for: Full-length genome sequences of porcine epidemic diarrhoea virus strain CV777; Use of NGS to analyse genomic and sub-genomic RNAs
Source: PLoS One. 2018 Mar 1;13(3):e0193682. doi: 10.1371/journal.pone.0193682 (PMC5832266; doi:10.1371/journal.pone.0193682)
Supplement: S2 Table — (DOCX) [file pone.0193682.s002.docx]

**Supplementary Information for Rasmussen et al., Full-length genome sequences of porcine epidemic diarrhoea virus strain CV777; use of NGS to analyse genomic and sub-genomic RNAs.**

**S2 Table.**

Homogenous nt differences in WBR CV777 sequence compared to reference CV777 sequence (in 100% of the NGS reads)

| Nt position | Number of total reads | Number of read differences | Percentage | Reference  Seq nt | Identified  nt |
| --- | --- | --- | --- | --- | --- |
| 3438* | >100 | >100 | 100% | G | C |
| 5170* | >100 | >100 | 100% | A | G |
| 25713* | 77 | 77 | 100% | T | C |
| 21213 | 21 | 21 | 100% | A | G |
| 23819 | 13 | 13 | 100% | C | T |
| 26169 | 9 | 9 | 100% | G | T |
| 23884 | 7 | 7 | 100% | C | A |
| 19047 | 2 | 2 | 100% | A | G |
| 15005 | 1 | 1 | 100% | C | T |

*These changes are indicated in Table 1 as they are fully supported by >25 separate reads.
