# Supplementary material for: Full-length genome sequences of porcine epidemic diarrhoea virus strain CV777; Use of NGS to analyse genomic and sub-genomic RNAs
Source: PLoS One. 2018 Mar 1;13(3):e0193682. doi: 10.1371/journal.pone.0193682 (PMC5832266; doi:10.1371/journal.pone.0193682)
Supplement: S3 Table — (DOCX) [file pone.0193682.s003.docx]

**Supplementary Information for Rasmussen et al., Full-length genome sequences of porcine epidemic diarrhoea virus strain CV777; use of NGS to analyse genomic and sub-genomic RNAs.**

**Table S3.**

Heterogeneous sequence differences in WBR CV777 sequence compared to reference CV777 sequence (heterogeneity present in >33% and ≤50% of the NGS reads)

| Nt position | Number of total reads | Number of read differences | Percentage | Reference  Seq nt | | Identified nt |
| --- | --- | --- | --- | --- | --- | --- |
| 3533 | 70 | 29 | 41% | A | | T |
| 27480 | 40 | 14 | 35% | A | | 12xG/2xT |
| 1557 | 8 | 4 | 50% | T | | A |
| 1555 | 8 | 4 | 50% | T | | A |
| 25083 | 6 | 2 | 33% | T | | C |
| 24129 | 5 | 2 | 40% | T | | G |
| 24126 | 4 | 2 | 50% | T | | G |
| 23997 | 5 | 2 | 40% | C | | A |
| 18389 | 4 | 2 | 50% | A | | G |
| 12863 | 4 | 2 | 50% | A | | G |
|  |  |  |  | |  |  |
