# Supplementary material for: Full-length genome sequences of porcine epidemic diarrhoea virus strain CV777; Use of NGS to analyse genomic and sub-genomic RNAs
Source: PLoS One. 2018 Mar 1;13(3):e0193682. doi: 10.1371/journal.pone.0193682 (PMC5832266; doi:10.1371/journal.pone.0193682)
Supplement: S4 Table — For each preparation the total collection of reads were mapped separately to the Br1/87 leader/TRS-L and the CV777 leader/TRS-L. (DOCX) [file pone.0193682.s004.docx]

**Supplementary Information for Rasmussen et al., Full-length genome sequences of porcine epidemic diarrhoea virus strain CV777; use of NGS to analyse genomic and sub-genomic RNAs.**

**Table S4.**

Number of read variants (with 100% identity) present in two independent Br1/87 RNA preparations from virus-infected cells made at 24h and 48h post infection (hpi). For each preparation the total collection of reads were mapped separately to the consensus Br1/87 leader/TRS-L and the reference CV777 leader/TRS-L sequences.

| Region | Br1/87 variant | | CV777 variant | |
| --- | --- | --- | --- | --- |
|  | No. of Reads  24 hpi | No. of Reads  48 hpi | No. of Reads  24 hpi | No. of Reads  48 hpi |
| Leader/TRS-L/genome | 74 | 119 | 8 | 28 |
| Leader/TRS-L/S | 35 | 91 | - | 2 |
| Leader/TRS-L/ORF3 | 5 | 14 | 5 | 6 |
| Leader/TRS-L/E | - | 6 | - | 4 |
| Leader/TRS-L/M | 11 | 19 | 4 | 7 |
| Leader/TRS-L/N | 26 | 37 | 17 | 29 |
| Total no. of reads | 151 | 286 | 34 | 76 |
