# Supplementary material for: Full-length genome sequences of porcine epidemic diarrhoea virus strain CV777; Use of NGS to analyse genomic and sub-genomic RNAs
Source: PLoS One. 2018 Mar 1;13(3):e0193682. doi: 10.1371/journal.pone.0193682 (PMC5832266; doi:10.1371/journal.pone.0193682)
Supplement: S5 Table — (DOCX) [file pone.0193682.s005.docx]

**Supplementary Information for Rasmussen et al., Full-length genome sequences of porcine epidemic diarrhoea virus strain CV777; use of NGS to analyse genomic and sub-genomic RNAs.**

**S5 Table**.

Oligonucleotide primers used for the production of overlapping amplicons at IZSLER.

| Primers | Oligonucleotide Sequence (5'-3') | Nt Position* |
| --- | --- | --- |
| 67_1_for | ATCTAYGGATAGTTAGCTCT | 18-37 |
| PEDV_2014_1_rev | TCCAATTTGTTGTCCATAAGTA | 5255-5276 |
| PEDV_2014_2_for | GATGTGGAGCGTTTCTAC | 5163-5180 |
| PEDV_2014_2_rev | ATTATGAACAGCCCACTCA | 9543-9560 |
| PEDV_2014_3_for | GTAGCGACTTAGATGGTG | 9805-9822 |
| PEDV_2014_3_rev | ATGTGGTGTGCTTAGAGC | 14496-14513 |
| 67_7_for | AGATTTCTTCCGTTCAGTCTA | 10995-11015 |
| 67_9_rev_bis | CATCATAACACTCAACACGA | 16393-16412 |
| 67_9_for | CAAGGAGGAGAGCGTTA | 15796-15812 |
| 67_9_rev_tris | GAAGGCATGGAATAACCAC | 19752-19770 |
| 67_10_for_bis | GACGATTTTGTCAGCATTCT | 19595-19614 |
| 67_11_rev_bis | GTGACCACACAACTCTCAAT | 24295-24314 |
| PEDV_2014_5_for | CTAATGTGCTGGGTGTTTC​ | 23252-23266 |
| 67_12_rev_tris | GTATCCATATCAACACCGTC | 28012-28031 |
|  |  |  |
| *nucleotide position for the complete genome of the reference sequence for PEDV CV777 (AF353511) | |  |
